# Evaluation of Nucleoside Analogs as Antimicrobials Targeting Unique Enzymes in *Borrelia burgdorferi*

**DOI:** 10.3390/pathogens9090678

**Published:** 2020-08-20

**Authors:** Monideep Chakraborti, Samantha Schlachter, Shekerah Primus, Julie Wagner, Brandi Sweet, Zoey Carr, Kenneth A. Cornell, Nikhat Parveen

**Affiliations:** 1 Department of Microbiology, Biochemistry and Molecular Genetics, Rutgers New Jersey Medical School, Newark, NJ 07103, USA; monideep2255@gmail.com (M.C.); sschlachter@steu.edu (S.S.); Shekerah@gmail.com (S.P.); 2 Department of Biology, Saint Elizabeth University, 2 Convent Road, Henderson Hall Room 112C, Morristown, NJ 07960, USA; 3 Department of Chemistry and Biochemistry, Boise State University, Boise, ID 83725, USA; Juliewagner@boisestate.edu (J.W.); Brandisweet@boisestate.edu (B.S.); Zoeycarr@boisestate.edu (Z.C.); Kencornell@boisestate.edu (K.A.C.); 4 Bridges to Baccalaureate Program, Boise State University, Boise, ID 83725, USA; 5 Biomolecular Research Center; Boise State University, Boise, ID 83725, USA

**Keywords:** methylthioadenosine/S-adenosylhomocysteine nucleosidase, *Borrelia burgdorferi*, antimicrobials, Bgp, Pfs, MTAN

## Abstract

The first line therapy for Lyme disease is treatment with doxycycline, amoxicillin, or cefuroxime. In endemic regions, the persistence of symptoms in many patients after completion of antibiotic treatment remains a major healthcare concern. The causative agent of Lyme disease is a spirochete, *Borrelia burgdorferi*, an extreme auxotroph that cannot exist under free-living conditions and depends upon the tick vector and mammalian hosts to fulfill its nutritional needs. Despite lacking all major biosynthetic pathways, *B. burgdorferi* uniquely possesses three homologous and functional methylthioadenosine/S-adenosylhomocysteine nucleosidases (MTANs: Bgp, MtnN, and Pfs) involved in methionine and purine salvage, underscoring the critical role these enzymes play in the life cycle of the spirochete. At least one MTAN, Bgp, is exceptional in its presence on the surface of Lyme spirochetes and its dual functionality in nutrient salvage and glycosaminoglycan binding involved in host-cell adherence. Thus, MTANs offer highly promising targets for discovery of new antimicrobials. Here we report on our studies to evaluate five nucleoside analogs for MTAN inhibitory activity, and cytotoxic or cytostatic effects on a bioluminescently engineered strain of *B. burgdorferi*. All five compounds were either alternate substrates and/or inhibitors of MTAN activity, and reduced *B. burgdorferi* growth. Two inhibitors: 5′-deoxy-5′-iodoadenosine (IADO) and 5′-deoxy-5′-ethyl-immucillin A (dEt-ImmA) showed bactericidal activity. Thus, these inhibitors exhibit high promise and form the foundation for development of novel and effective antimicrobials to treat Lyme disease.

## 1. Introduction

Lyme disease is the most prevalent tick-borne illness in the USA, with a reported incidence rate of over 300,000 cases annually [1]. *Borrelia burgdorferi* sensu stricto is the primary cause of Lyme disease in North America, while different *Borrelia* species including *Borrelia garinii* and *Borrelia afzelii* cause borreliosis in Europe [2,3]. Infection by these spirochetes is conventionally treated by monotherapy with doxycycline, amoxicillin, or cefuroxime axetil as the first line therapy [4,5,6]. Ceftriaxone is used for the late Lyme disease, particularly when there are neurological manifestations, or to treat patients for whom tetracycline or penicillin cannot be used [7,8,9,10]. Treatment of penicillin-sensitive and pregnant women with doxycycline also remains a problem. Macrolides are recommended only for pregnant women and patients intolerant to the first-line therapy. A significant subset of patients continues to show subjective symptoms such as pain, fatigue, or neurological complications after completion of the antibiotic treatment regimen. Although drug resistance in *B. burgdorferi* is not as rampant as in Gram-negative and Gram-positive bacteria, Lyme spirochetes show relatively high tolerance to aminoglycosides and some macrolides, for example erythromycin, such that they are not effective in Lyme disease treatment as the first line therapeutics. Thus, discovery of novel targets in *B. burgdorferi* and effective new drugs that function as stand-alone therapies or are useful in combination drug regimens will benefit increasing numbers of patients suffering from this disease.

Bacterial 5′-methylthioadenosine/S-adenosylhomocysteine nucleosidase (MTAN) has four known substrates: (i) S-adenosylhomocysteine (SAH), which is cleaved to produce S-ribosylhomocysteine and adenine, (ii) 5′-methylthioadenosine (MTA) that is hydrolyzed to 5-methylthioribose (MTR) and adenine, (iii) 5′-deoxyadenosine (5′dADO) that is degraded to 5-deoxyribose and adenine, and (iv) 6-amino-6-deoxy-futalosine, which is produced in some organisms expressing the menaquinone pathway [11,12,13,14,15,16,17,18]. Although detoxification of these growth inhibitory nucleosides is a major role of MTAN in bacteria, its contribution is also important in autoinducer-1 and autoinducer-2 mediated quorum sensing mechanisms that trigger the expression of virulence factors during infection in numerous Gram-negative bacterial pathogens [19]. Finally, this enzyme plays important roles in methionine and purine recycling from byproducts of methylation reactions, polyamine synthesis and quorum sensing mechanisms in Gram-negative and Gram-positive bacteria that ultimately impact bacterial fitness through the salvage of nutrients that are metabolically expensive to synthesize de novo [11,19].

Most bacteria contain only a single gene that encodes a cytoplasmic MTAN. *B. burgdorferi* is the only known organism to possess three MTANs: chromosomally localized *bb0375* (Pfs) and *bb0588* (for Borrelia Glycosaminoglycan binding Protein, Bgp) genes and the plasmid encoded *bbi06* gene (MtnN). Interestingly, Bgp and MtnN possess signal peptides indicating that these proteins are exported through the cytoplasmic membrane. We have previously demonstrated that Bgp is indeed present on the surface of the spirochetes [20]. Furthermore, our extensive studies have shown that Bgp contains both adhesin and MTAN functions in the spirochete [20,21], and both Bgp and Pfs are known to be expressed during host infection [22]. All three proteins display MTAN activities and hydrolyze the native nucleoside substrates, although with varying degrees of efficiency [18,21]. Retention of all three genes in different strains of *B. burgdorferi* [23], which is an extreme auxotroph that has lost most of the genes of biosynthetic pathways involved in producing building blocks of macromolecules, suggests the roles of MTANs are critical for survival of this spirochete species. It is likely that MTANs also play important roles in recycling adenine and methionine to supplement the spirochete’s need for these essential nutrients in the nutritionally limiting environments they encounter during infection of the mammalian hosts and during colonization of tick vector [11]. We previously reported that *bgp*-defective mutants of two *B. burgdorferi* strains showed attenuated virulence in a mouse model of Lyme disease, further emphasizing the critical role of this protein during infection [24,25].

Although MTAN is primarily a bacterial enzyme that does not exist in eukaryotes, MTAN encoding genes have been found in the genomes of several unicellular eukaryotic pathogens, including *Giardia intestinalis, Entamoeba histolytica,* and *Trichomonas vaginalis.* Interestingly, similar to Lyme spirochetes [11], these organisms are strict purine auxotrophs that lack de novo purine synthetic pathways. In addition, adenine nucleotides are important mediators of cellular homeostasis that universally couple cellular metabolism, DNA or RNA synthesis, and other physiological processes [26,27]. During infection, ATP, ADP, and adenosine also play a role in the damage-associated molecular patterns used by the host to activate the immune system [28]. It has been suggested that differences between patients with respect to the extracellular nucleotide concentrations may contribute to variable antibiotic treatment outcomes during infection [29].

**Figure 1:**
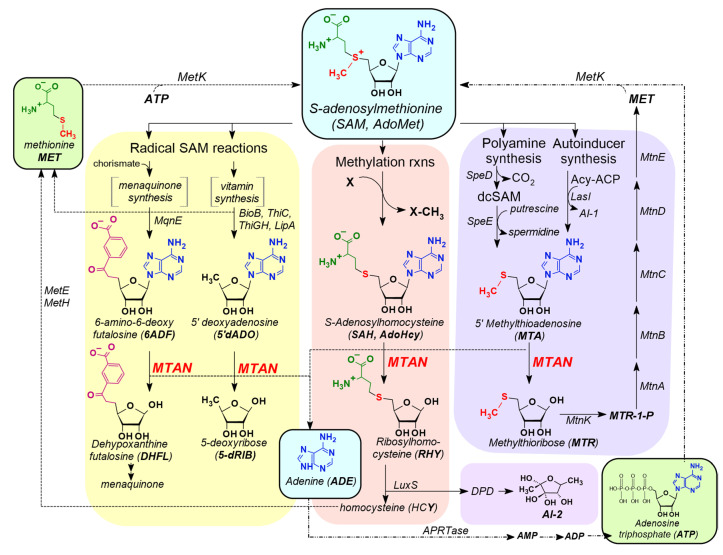
Bacterial metabolic pathways showing the involvement of MTA/SAH nucleosidase (MTAN). Conversion of the substrates: MTA, SAH, 5′dADO and 6ADF by MTANs into respective byproducts is shown. Inhibition of MTA/SAH nucleosidase will prevent this cascade of biochemical reactions leading to growth inhibition.

As such, MTANs of *B. burgdorferi* may serve as a model to study the druggability of this enzyme in various pathogens that are purine auxotrophs. In both bacteria and mammalian cells, SAH is a potent feedback inhibitor of S-adenosylmethionine (SAM)-dependent methylation reactions, while MTA acts as a feedback inhibitor of polyamine synthases, and 5′dADO inhibits radical SAM reactions [30]. Thus, inhibition of MTAN activity likely causes an accumulation of MTA, SAH, and 5′dADO within bacterial cells, which can have wide-ranging physiological consequences (Figure 1) [31]. The limitation of polyamine availability influences DNA replication, resulting in growth arrest of microorganisms. The buildup of 5′dADO produced from radical SAM reactions reduces vitamin availability required for numerous metabolic reactions and probably alters flux rates through central glycolytic pathways, causing reduced cellular growth [13]. An *Escherichia* *coli* MTAN knockout strain was reported to accumulate SAH to more than 50 μM, a concentration that was approximately 50-fold greater than that of the isogenic wild-type strain, further supporting critical roles of MTAN even in prototrophic bacteria. The study also showed that SAM, SRH, and methionine levels decreased in the knockout strain, potentially due to the loss of the salvage pathway [32]. It is expected that these outcomes are further exacerbated in auxotrophic bacteria that may depend on salvage pathways for their survival making them ideal drug targets. Therefore, the presence of multiple MTANs in *B. burgdorferi* makes them a useful model for polypharmacological studies [33], and for discovery of new antibiotics for spirochetes and other organisms [34], particularly purine auxotrophs.

Although fluorescence has been the most widely used chemistry in life science research, bioluminescence has become more popular than fluorescence for various types of analyses over the last decade [35]. Bioluminescence has proved to be beneficial in the investigation of novel antimicrobials against bacterial pathogens in a non-invasive manner [36,37,38,39,40]. Bioluminescence is a natural process and can have up to 1000-fold higher sensitivity than fluorescence-based assays [41]. Fluorescence emission in assays is really bright due to exposure to the high rate of photons, which can also lead to higher backgrounds. The bioluminescence-based assays may not emit light as bright as in fluorescence assays, but background bioluminescence is almost absent [35]. Additionally, in bioluminescence, the light emitting ability is a natural part of the biological system, which supports the energy transition process for maximum yield [41]. The role of any enzyme is to accelerate the conversion of substrate into its products. Luciferase activity results in metabolism of the D-luciferin substrate with some of energy released in the form of light emission [35]. Oxygen and ATP are used in order to carry out this chemical reaction [35,42]. Thus, bioluminescence is associated with vitality of the living organisms.

**Figure 2:**
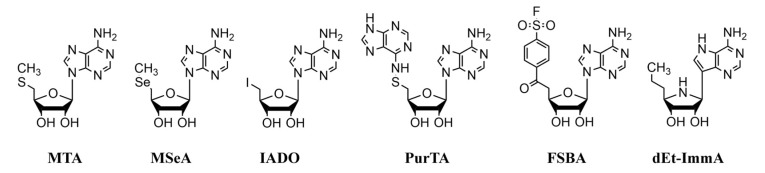
Structures of substrates and nucleoside analog inhibitors of *Borrelia* MTANs. **MTA** = 5′-deoxy-5′-methylthioadenosine, **MSeA** = 5′-deoxy-5′-methylselenoadenosine, **IADO** = 5′-deoxy-5′-iodoadenosine, **PurTA** = 5′-deoxy-5′-purinylthioadenosine, and **FSBA** = 5′-(fluorosulfonylbenzoyl)adenosine **dEt-ImmA** = 5′-deoxy-5′-ethyl-immucillin A.

The availability of a bioluminescent *B. burgdorferi* strain generated in our laboratory [43] offered us a unique opportunity to use luciferase enzyme activity levels as an indication of spirochete viability following treatment with the different proposed antibiotics. In this study, we evaluated five nucleosides/nucleoside analogs: 5′-deoxy-5′-methylselenoadenosine (MSeA), 5′-deoxy-5′-iodoadenosine (IADO), 5′-(fluorosulfonylbenzoyl)adenosine (FSBA), 5′-deoxy-5′-purinylthioadenosine (PurTA), and the non-hydrolyzable transition state analog 5′-deoxy-5′-ethyl-immucillin A (dEt-ImmA) (Figure 2) for activity against *Borrelia* MTAN enzymes, and further studied their bacteriostatic and bactericidal effects against a bioluminescent derivative of *B. burgdorferi* strain N40 D10/E9. Among the five compounds tested, we found that the competitive inhibitors, IADO and dEt-ImmA, showed the most potential, since they exerted low nanomolar to low micromolar inhibition constants against the *B. burgdorferi* MTANs, and produced bactericidal activity against *B. burgdorferi* culture. Thus, these nucleoside analogs exhibit high promise as lead compounds to design effective antimicrobials to treat Lyme disease.

## 2. Materials and Methods

### 2.1. Ethics Statement

Experiments with *B. burgdorferi* were conducted in Parveen’s laboratory at the New Jersey Medical School (NJMS) under her approved Institutional Biosafety Level 2 protocol to work with this pathogen. Recombinant protein expression and enzyme analysis were conducted in the Cornell lab under approved BSU IBC protocols.

### 2.2. B. burgdorferi Culture

*B. burgdorferi* N40 strain carrying a firefly luciferase gene (Bbluc) [43], which is a derivative of the N40D10/E9 clone [44,45], was used in this study and is labeled as N40 throughout. N40 was cultured at 33 °C in Barbour-Stoenner-Kelly-II (BSK-II) medium supplemented with 6% rabbit serum (BSKII-RS). Bacterial counts were determined under 400× magnification using a hemocytometer and a dark field microscope.

### 2.3. Substrates and Inhibitors

The nucleosides 5′-deoxy-5′-methylthioadenosine (MTA), 5′-(fluorosulfonylbenzoyl)-adenosine (FSBA) and 5′-deoxy-5′-iodoadenosine (IADO) were obtained from Sigma Chemical Co. (St. Louis, MO, USA). The nucleosides 5′-deoxy-5′-purinylthioadenosine (PurTA), and 5′-deoxy-5′-methylselenoadenosine (MSeA) were the kind gift of Dr. Michael Riscoe (Portland VA Medical Center, Portland, OR, USA). The nonhydrolyzable transition state analog 5′-deoxy-5′-ethyl-immucillin A (dEt-ImmA) was generously provided by Dr. Y.S. Babu (BioCryst, Birmingham, AL, USA).

### 2.4. Expression and Purification of Recombinant MTANs

Recombinant MTANs were expressed and purified as previously described [18]. Briefly, overnight 10 mL cultures of recombinant *E. coli* BL21(DE3) pLysS cells containing engineered pET30a expression plasmids for Bgp, MtnN or Pfs were grown at 37 °C with shaking (150 rpm) in LB broth supplemented with 50 µg/mL kanamycin and 0.5% glucose. The culture was diluted 1:50 in fresh medium and incubated at 37 °C with shaking (150 rpm) until the absorbance (600 nm) reached 0.4–0.6. Protein expression was induced with the addition of isopropyl β-D-1-thiogalactopyranoside (IPTG) to a final concentration of 1 mM and continued overnight incubation with shaking at 30 °C. Cells were harvested by centrifugation (10,000× *g*/15 min), and the pellet was washed once with ice-cold lysis buffer (50 mM sodium phosphate, pH = 8.0, 300 mM NaCl, 10 mM imidazole, 2mM β mercaptoethanol). Harvested cell pellets were resuspended in 10 mL fresh lysis buffer and lysed by sonication on ice using a Misonix Sonicator 300 (power setting 7, pulse 30 s, 1 min cooling with total sonication for 2.5 min). The cell lysates were centrifuged at 10,000× *g* for 15 min at 4 °C to remove debris, and the lysate was further filtered through a 0.45 μM syringe filter to obtain a clear extract. Recombinant N-terminal hexahistidine tagged proteins were purified from the lysate using HisPur™ affinity columns (Thermo Scientific) according to the manufacturer’s specifications, and specifically eluted with 250 mM imidazole containing buffer (pH = 7). Eluted materials were analyzed by SDS-PAGE and Coomassie blue staining and found to be >95% homogeneous. Enzyme concentrations were determined using UV scans on a Cary100 spectrophotometer (Agilent) to determine the absorbance at 280 nm and applying extinction coefficients (0.1%) of 0.594 (Bgp), 0.637 (MtnN), and 0.404 (Pfs) [18]. Enzyme stocks (1 mg/mL) were prepared in 20% glycerol and stored at −80 °C.

### 2.5. Enzyme Activity and Kinetics

Enzyme specific activities were determined using previously reported optimal pH conditions (50 mM sodium phosphate, pH = 7, for Bgp, MtnN; 50 mM sodium phosphate-citrate, pH = 5, for Pfs) and a spectrophotometric activity assay that measures the decrease in UV absorbance at 275 nm that occurs when MTA is cleaved to 5-methylthioribose and adenine (ε_275_ = 1.6 mM^−1^cm^−1^) [18,46]. The extinction coefficients for cleavage of other nucleosides were experimentally validated and found to be the same for MSeA, FSBA, and IADO (ε_275_ = 1.6 mM^−1^cm^−1^). Due to the added UV absorbance imparted by the 5′ purine group found in PurTA, the enzyme reactions were followed spectrophotometrically at 260 nm using the experimentally determined extinction coefficient of ε_260_ = 5.6 mM^−1^cm^−1^. Enzyme specific activities for MTA were generally 1–2 U/mg (1 U = µmol MTA hydrolyzed/min) [18]. The enzyme specific activities for other nucleosides were determined and reported in U/mg and as the % max specific activity (relative to MTA at 100%).

Kinetic constants have been previously reported for MTA [18]. As MSeA was also an efficient substrate for the *B. burgdorferi* nucleosidases, kinetic constants were determined using the activity assay described above, varying the nucleoside concentrations from 1 to 20 µM, and fitting the data on substrate-velocity plots to the Michaelis–Menten equation using GraphPad Prism (version 6):

(1) $$Vo=\frac{Vmax\left[S\right]}{Km+\left[S\right]}$$

For the other compounds, inhibition constants (Ki’s) were determined as previously described [18,46], using an MTA concentration of 100 µM and varying the inhibitor concentration from 0 to 100 µM. The inhibited velocity (Vo’) and uninhibited velocity (Vo) values were determined, and the ratio of Vo’/Vo plotted as a function of inhibitor concentration. Ki values were calculated from these plots by fitting the data to the equation for competitive inhibition using GraphPad Prism:

(2) $$\frac{Vo^{\prime }}{Vo}=\frac{Km+\left[S\right]}{Km+\left[S\right]+\left(\frac{Km\left[I\right]}{Ki}\right)}\;\;$$

The enzyme inhibition constant and delayed onset inhibition constant for dET-ImmA was previously reported [18].

### 2.6. Luciferase Assay for B. burgdorferi Survival

The luciferase expressing *B. burgdorferi* N40 strain was grown to a density of ~10^8^ spirochetes/mL and divided into two aliquots. One aliquot was incubated at 60 °C for 30 min to kill the spirochetes. A 10-fold serial dilution of dead *B. burgdorferi* was prepared in the second aliquot of live bacterial suspension such that the ratio of live spirochetes decreased from 100% to 0% and dead spirochetes increased from 0% to 100% (i.e., 100:0 to a final 0:100 ratio of live:dead *B. burgdorferi*). Two hundred microliters of culture mixture were transferred to each well of a 96-well black plate with a clear bottom (catalog number 3603, Corning Incorporated, New York, NY, USA). For the luciferase activity determination (bioluminescence assay), the plate was kept on ice and 4 µL of D-luciferin substrate (30 mg/mL) was added to each well. The culture was mixed with the substrate by shaking in the dark, incubated at 37 °C for 5 min in a Spectramax M2 microplate reader (Molecular Devices, San Jose, CA, USA) and the bioluminescence measured. Three replicates were used for each culture and each experiment was repeated at least three times to confirm reproducibility of the assay.

### 2.7. Assessment of Nucleosides/Nucleoside Analogs as Antimicrobials

Nucleosides and nucleoside analogs were prepared as sterile 1 mM stock solutions in distilled water (MSeA, IADO, FSBA, and dEt-ImmA) or in DMSO (PurTA). Treatment with the inhibitors was carried out for a period of 7 days. The N40 culture was grown to 2 × 10^8^ spirochetes/mL. Six 5-fold serial dilutions of 1 mM stock solutions of each inhibitor were prepared in 1.5 mL Eppendorf tubes to a final concentration of 0.0032–10 µM. Briefly, 10 µL of inhibitor was added to 990 µL of culture (10 µM final concentration) in the first tube and vortexed. Fivefold serial dilutions were prepared by adding 200 µL of this inhibitor containing culture to 800 µL of fresh culture. Untreated *B. burgdorferi* culture controls were included to show normal baseline bioluminescence levels. Photon emission due to luciferase activity was measured at three time points, after: (a) 24 h, (b) 72 h, and (c) 7 days of treatments to observe the effect of inhibitors on *B. burgdorferi* culture. At the end of each time point, bioluminescence was measured as described above. Three replicates were used for each treatment and each experiment was repeated at least two times to confirm reproducibility of results. The standard prepared from the live:dead *B. burgdorferi* experiment was used to calculate the percentage of dead spirochetes in each treated sample based upon bioluminescence levels.

### 2.8. Assessment of Bacteriostatic Versus Bactericidal Activities of Nucleosides and Nucleoside Analogs

After 7 days of treatment, a 5 µL aliquot from each treatment tube (and untreated control) was added to 1 mL of BSKII-RS medium to allow any remaining viable spirochetes in the sample to grow. After incubation for 7–21 days at 33 °C, the survival of the spirochetes was determined using dark-field microscopy to observe motility and by employing bioluminescence measurements to assess vitality of the recovered culture.

## 3. Results

### 3.1. MTAN is a Critical Enzyme in the Salvage Pathways of Auxotrophic Bacteria

A linear correlation between faster bacterial growth rate and susceptibility to β-lactam antibiotics has been reported [47]. Based upon that report, it is possible that tolerance of *B. burgdorferi* for many antibiotics could be due to its slow growth rate. In fact, the metabolic basis for antibiotic-mediated cell death has implications for the development of MTAN inhibitors as drugs to treat Lyme spirochetes (Figure 1). Thus, inhibition of all three MTANs by the same inhibitors could enhance their effect as antimicrobials, particularly because uptake of the inhibitors may not be required due to localization of Bgp and MtnN enzymes on or near the surface of the spirochete.

### 3.2. Kinetic Analysis of Purified Recombinant MTANs with Nucleosides and Nucleoside Analogs

The relative specific activity measurements for the three MTANs for the four nucleosides MSeA, IADO, PurTA, and FSBA are presented in Table 1. Interestingly, both Bgp and MtnN readily hydrolyzed MSeA with specific activities that were 50–79% higher than MTA, indicating that the substitution of a sulfur atom by selenium did not negatively impact the hydrolysis of this nucleoside. However, MSeA was only a modest substrate for Pfs, showing only 16% of the activity of MTA. The remaining nucleosides (IADO, PurTA, and FSBA) were poor substrates for all of the enzymes, particularly for MtnN and Pfs.

The Michaelis constant (Km) was determined from substrate-velocity plots of MSeA data (summarized in Table 2). All three nucleosidases show similar recognition of MSeA, with Km values of 1.5–2.1 µM. For Bgp and Pfs, this Km is approximately 3-fold higher than reported for MTA. Inhibition constants were determined for the remaining three nucleoside analogs (IADO, PurTA, FSBA) and the nonhydrolyzable nucleoside analog (dEt-ImmA). Pfs was most susceptible to inhibition by nucleoside analogs, with sub-micromolar Ki values. MtnN showed the least affinity for nucleoside inhibitors (Ki values of 2–2.5 µM). Overall, it is clear that the nucleosides, while being poor catalytic substrates for the MTANs, were still well recognized by the enzyme active sites. Lastly, the nonhydrolyzable transition state analog (dET-ImmA) was the best inhibitor and showed added slow-onset inhibition (Ki’) [18,46]. The affinity of dET-ImmA for MTANs is approximately 1000-fold tighter than the other nucleosides, with Ki and Ki’ values that are in the low nanomolar to sub-nanomolar concentration range.

### 3.3. Determine Efficacy of Luciferase Activity to Measure B. burgdorferi Viability

The correlation between bioluminescence and N40 spirochete cell number was determined using three different concentrations of *B. burgdorferi* (Table 3). Bioluminescence was measured for 1 × 10^8^ spirochetes/mL, 1.5 × 10^8^ spirochetes/mL, and 2 × 10^8^ spirochetes/mL. In all cases, a direct correlation was observed between bioluminescence levels and live spirochete numbers, with the highest coefficient of correlation (R^2^ = 0.9834) observed when the *B. burgdorferi* culture density was 2 × 10^8^ spirochetes/mL.

**Figure 3:**
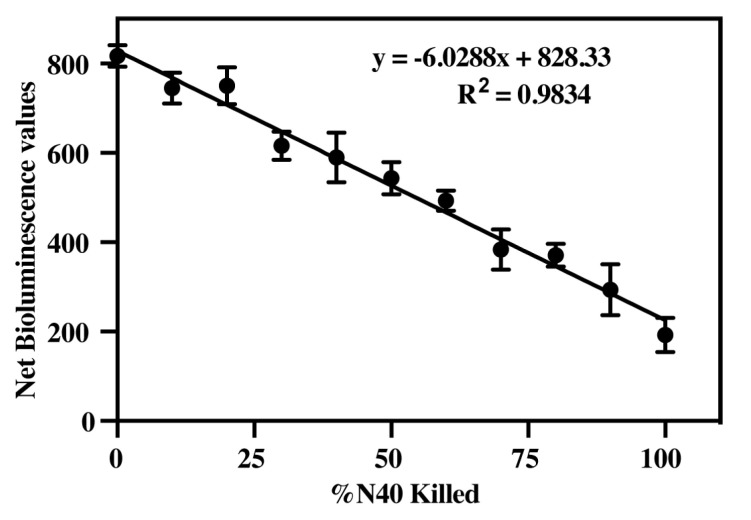
Standard curve showing loss of bioluminescence with percentage of heat killed infectious N40 spirochetes. The standard curve using 2 × 10^8^ spirochetes/mL showed a high coefficient of correlation (R^2^ = 0.9834) of bioluminescence levels with live bacteria such that heat treatment resulted in the loss of luciferase activity and resulting bioluminescence levels.

As expected in the live:dead *B. burgdorferi* experiment, light emission diminished as the percentage of dead *B. burgdorferi* increased in the mixture. A standard curve was prepared using the mean luciferase activity readings with respect to percent of live spirochetes present in live and dead *B. burgdorferi* cultures mixtures (Figure 3). Thus, we could determine the percentage of physiologically compromised *B. burgdorferi* based upon the respective bioluminescence values. The high correlation coefficient with physiologically compromised bacteria suggested that when the vitality of spirochetes was affected, the corresponding drop in intracellular ATP concentration limits luciferase activity and the concomitant bioluminescent output. The lower sensitivity of detection of luciferase activity was limited to approximately 10% live spirochetes (~10^7^ *B. burgdorferi* cells/mL or 2 × 10^6^ spirochetes/well). These results indicated that highly active spirochete density can be determined accurately by measurement of bioluminescence. We further used this standard for determination of the effectiveness of ampicillin and the nucleosidase inhibitors in killing spirochetes.

### 3.4. Effect of Ampicillin and Selected Inhibitors of MTANs on the Growth of B. burgdorferi

**Figure 4:**
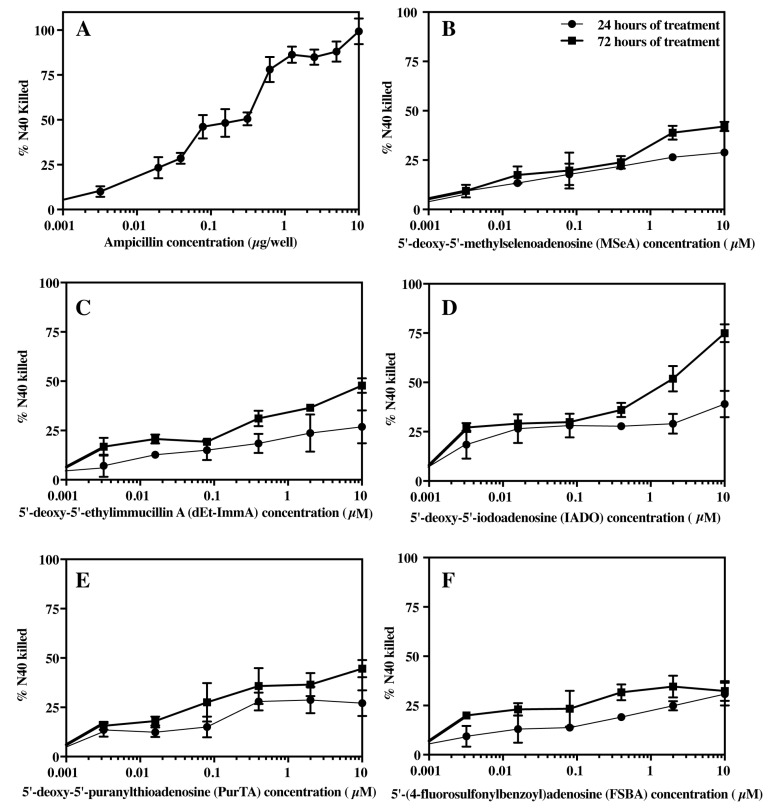
Bioluminescence-based evaluation of anti-*B. burgdorferi* activities of ampicillin and five MTAN inhibitors using *B. burgdorferi* strain N40. (**A**) The standard curve was used to determine the percent of *B. burgdorferi* killed after treatment with different concentrations of ampicillin (µg/well) for 24 h. Loss of *B. burgdorferi* vitality was measured by bioluminescence assay after treatment with MTAN inhibitors for 24 h (closed circles) and 72 h (closed squares): (**B**) MSeA, (**C**) dEt-ImmA, (**D**) IADO, (**E**) PurTA, and (**F**) FSBA for 24 h and 72 h. The standard curve was used to determine the percent of dead/physiologically inactive spirochetes of *B. burgdorferi* after treatment with different concentrations of inhibitors (µM). All the inhibitors showed a gradual increase in spirochete killing over the course of the treatment when compared with ampicillin and untreated controls.

The infectious N40 *B. burgdorferi* culture was treated with ten 2-fold serial dilutions of ampicillin overnight. Stocks of ampicillin at 200 µg/mL to 0.385 µg/mL concentrations were used to prepare different dilutions for treatment (final 10 to 0.0195 µg/well). The bioluminescence standard curve (Figure 3) was used to calculate the percentage of physiologically compromised *B. burgdorferi* after treatment with different ampicillin concentration for 24 h. Ampicillin treatment affected the viability of the spirochetes (Figure 4A). The IC_50_ for ampicillin was calculated from graphs and determined to be ~0.31 µg/µL of culture. A prolonged incubation could increase killing by ampicillin at lower doses of drug. In addition to bioluminescence, the cultures were also observed under dark-field microscopy to examine viability. At ≥0.625 µg/well concentrations, majority of the spirochetes were non-motile and showed stripping of the outer membrane and the formation of vesicles. A few spirochetes showed motility when the concentration of ampicillin was less than 0.625 µg/well during the 24hr treatment.

The inhibitory activities of the five compounds against the infectious N40 *B. burgdorferi* culture were determined over a period of seven days. Bioluminescence was measured at three time points after inhibitor treatment for (A) 24 h, (B) 72 h, and (C) 7 days (Table 4). The MTAN inhibitors only partially affected the physiological status of *B. burgdorferi* after 24 h of treatment, even at high concentrations (10 µM). This is not necessarily surprising because of slow growth rate of *B. burgdorferi*. Bioluminescence measurements after 72 h of treatment indicated the effectiveness of MTAN inhibitors to adversely alter spirochete viability, with a higher percent of N40 killing at this time point. Of all of the inhibitors, the nucleoside IADO was found to be the most effective in killing bacteria after 72 h of treatment. The IC_50_ values for the different inhibitors ranged from >10 µM for FSBA to ~1 µM for IADO.

Bioluminescence readings after seven days of incubation were similar to the negative control (100% dead spirochete culture) and are therefore not shown here. These results could be because (i) inhibitors affected metabolic activity of *B. burgdorferi* such that ATP presence diminished significantly that inhibited luciferase activity, (ii) the culture reached late stationary/death phase of growth phase of *B. burgdorferi* irrespective of treatment with the inhibitors, and (iii) the killing the bacteria by inhibitors was completed by this time point. These outcomes make the use of bioluminescence ideal for screening of novel antimicrobials. After seven days of treatment, physiologically compromised and dead bacteria were easily discernible microscopically.

### 3.5. Bacteriostatic or Bactericidal Activities of MTAN Inhibitors Against B. burgdorferi N40

To further differentiate between bacteriostatic versus bactericidal activities of MTAN inhibitors, we subcultured drug treated spirochetes in the absence of inhibitors to recover any live *B. burgdorferi* remaining after seven days of inhibitor treatment (summarized in Table 4). If non-motile spirochetes from treated cultures were able to grow back, the inhibitor was determined to have a bacteriostatic effect at the selected concentration. If the spirochetes failed to recover, the inhibitor was considered to exhibit a bactericidal effect at that specific inhibitor concentration. Spirochete survival was determined by both dark-field microscopy and bioluminescence measurements. Microscopic examination allowed assessment of motility of the spirochetes whereas bioluminescence assays assessed recovery and viability of *B. burgdorferi* cells. After 12 days of incubation of subcultured bacteria, bioluminescence levels were comparable with untreated controls, suggesting that the spirochetes fully recovered when they were grown in the absence of the inhibitors after treatment with all concentrations of PurTA, FSBA and MSeA. Thus, these inhibitors exhibit bacteriostatic rather than bactericidal activities against *B. burgdorferi,* even at the highest concentration tested in the study (10 µM) (Table 4). By comparison, a reduction in bioluminescence compared to the untreated control was observed after treatment with dEt-ImmA at a 0.4 µM or greater concentration. For the 2 µM and 10 µM dEt-ImmA and IADO concentrations, no live spirochetes were recovered, even after 21 days of subculture in media lacking MTAN inhibitors. These results indicate dEt-ImmA and IADO at concentrations ≥1 µM exert bactericidal activity against *B. burgdorferi* after seven days of treatment but were bacteriostatic at lower concentrations. After seven days of treatment, live spirochetes were only recovered when concentrations of ≤0.4 µM were used for dEt-ImmA and IADO.

## 4. Discussion

The first line of therapy for Lyme disease treatment primarily involves antibiotic classes such as penicillin, tetracycline, and cephalosporin. Sometimes, patients become sensitive to the first line of therapy. Macrolides like erythromycin are recommended in this scenario, but are not universally effective. Alternatively, a combination of drugs can be used for the treatment. Hence, there is an urgent need to discover new antimicrobials that are non-toxic, effective and will benefit those patients who are sensitive to the first line of therapy, such as pregnant women and potentially those suffering from chronic Lyme disease. Due to the critical role played by MTAN enzymes in purine and methionine auxotrophs, and because the enzyme is absent in humans [21], we assessed MTANs as target for discovery of novel antimicrobials against *B. burgdorferi*.

In most species of bacteria, MTAN catalyzes the irreversible decomposition of MTA, SAH, and 5′dADO to adenine and the corresponding sugar. In the menaquinone biosynthetic pathway expressed by some bacterial species (*Camplyobacter, Chlamydia,* and *Helicobacter*), the enzyme MqnE produces the nucleoside 6-amino-6-deoxy-futalosine from 5′dADO radical [16,17]. In some instances, MTAN is responsible for the hydrolysis of this nucleoside to adenine and dehypoxanthine futalosine. In each of these reactions, MTAN activity reduces the intracellular concentration of these growth inhibitory nucleosides, which are potent feedback inhibitors of the reactions that produce them. The enzyme also promotes the salvage of nutritionally valuable adenine and methionine that would otherwise be lost as a consequence of SAM dependent reactions. The ability of MTANs to recognize native nucleoside substrates with a variety of 5′-alkyl or 5′-alkylthio substitutions suggests that 5′-modifications will be tolerated in the design of competitive inhibitors. Potentially, these compounds would cause an inhibition of MTAN activity that would elevate the concentrations of MTA, SAH, 5′dADO, which would subsequently lead to growth inhibition through interruption of methylation reactions, polyamine synthesis, and radical SAM reactions that are involved in vitamin (thiamine, lipoate, and biotin) and menaquinone synthesis. These latter reactions have implications for vitamin dependent central carbon metabolism and energy production that would be expected to decrease growth rates and potentially lead to cytotoxic responses [11]. Alternatively, the loss of purine and methionine salvage may limit growth by depriving the cells of readily accessible nutrients. In the work described here, accumulation of toxic byproducts or reduced nutrient availability likely causes inhibition of growth of *B. burgdorferi*. The accompanying reduction in ATP synthesis and subsequent killing of the spirochetes is associated with reduction in the luciferase activity and subsequent emission of light in the bioluminescent *B. burgdorferi* strain used in these studies.

Humans lack MTAN, and instead use MTA phosphorylase (MTAP) and SAH hydrolase (SAHH) enzymes for catabolism of these nucleosides. Structurally and catalytically, there are distinct differences between the mammalian and bacterial enzymes that should be exploitable for drug design. For instance, MTAP has a smaller 5′alkylthio binding site when compared to bacterial MTAN [48,49,50,51,52,53], suggesting narrower substrate specificity [11]. Additionally, the 2′ hydroxyl recognition site is negatively charged in MTAN and positively charged in MTAP [11,50]. Based upon our threading of two *B. burgdorferi* Pfs and Bgp enzyme sequences on *E. coli* MTAN structure, difference between human MTAP and these two spirochete MTANs are expected to be comparable to the *E. coli* MTAN [21]. Furthermore, *B. burgdorferi* enzymes recognize and cleave SAH, and also show good specific activities for a series of alkylthio-analogs. In addition, butylthioDADMe-ImmA and HCY-ImmA proved to be potent inhibitors of these enzymes with nM to picomolar Ki’s [18]. Therefore, the active site appears sufficiently accessible for 5’ alkyl groups to bind, particularly with the conformational flexibility exhibited with straight chain or weakly branched alkyl groups. Since the specific activity measurements for IADO, FSBA, and PurTA indicate that these nucleosides are poorly hydrolyzed, but their corresponding Ki values are in the low micromolar to submicromolar concentration range (similar to the native substrate Km values for MTA and SAH). Our results indicate that these three nucleoside analogs are bound in the active site but are not readily catabolized. This suggests that the electronic structure of the 5′ substitution in these compounds decreases their ability to proceed through the oxocarbenium transition state that is a feature of the catalysis of MTA and SAH by nucleosidases [46]. These differences in enzymes may be beneficial for designing drugs against bacterial infections in humans.

Two MTANs, Bgp and MtnN of *B. burgdorferi,* are exported from the cytoplasmic membrane and are located on/near the spirochete surface [54] such that the cellular uptake of nucleosidase inhibitors may not be necessary for the drugs to have an inhibitory effect. Further, since the active sites of the *B. burgdorferi* MTANs are highly conserved, inhibitors would be expected to function across all three nucleosidases [21]. Because *B. burgdorferi* is an extreme auxotroph that lacks all major biosynthetic pathways, salvage pathways are potentially even more critical for recycling the purine adenine and the amino acid methionine to ensure spirochete growth and survival [21]. In addition, Yang and coworkers showed that purine biosynthesis is involved in antibiotic lethality and demonstrated that antibiotic-induced adenine limitation increases ATP demand and indirectly enhances the killing effect of antibiotics [55]. In the case of Lyme spirochetes, adenine limitation induced by MTAN inhibition could have a similar effect, causing increased ATP demand, decreased growth, and/or cytotoxicity. Therefore, the results reported here for MTAN inhibitors in the highly rich BSKII-RS medium could be expected to be even more pronounced during infection, since host environments are more nutritionally limited.

The enzyme inhibitory activity of the nucleosides MSeA and PurTA were previously tested against *E. coli* MTAN and reported to have similar low micromolar effects (~ 4 µM IC_50_s) using a radioactive enzyme assay [12]. In MSeA, selenium replaces the sulfur atom in the 5′-alkylthio moiety. Since selenium and sulfur are both group 6A elements with similar electronegativity, it is not surprising that MSeA was found to be both an efficient MTAN substrate and to show low micromolar Km values (1.5–2 µM) that approximate those for MTA (Table 2). Potentially, the observed bacteriostatic effects for this nucleoside are the consequence of it acting as a “pro-drug”, with subsequent metabolism to selenomethionine, adenosyl-selenomethionine, or other intermediates that ultimately have a negative effect on essential metabolic processes. For PurTA, a bulky purinylthio group replaces the 5′ methylthio group in MTA. While the Ki value for PurTA (~0.7–2 µM) indicates the compound binds tightly to the *B. burgdorferi* enzymes, the low specific activities (Table 1) for this compound suggest that catalysis is poor, possibly because the 5′ purinylthio group alters the electronic structure of the compound in a way that prevents it from proceeding through the transition state to allow electron withdrawal through the glycosidic bond to yield free adenine. Alternatively, the interactions with the 5′ alkylthio binding pocket may position the molecule sufficiently far away from catalytic residues in the active site to reduce the efficiency of the attack by the nucleophilic water molecule on C1 of the ribose. Similar to PurTA, FSBA contains a large, bulky 5′ alkyl group, and shows submicromolar to low micromolar Ki values for the *Borrelia* nucleosidases, but low specific activity. This indicates that FSBA binds well to the active site but is a poor target for catalysis. The structure of FSBA inhibitor is remarkably similar to 6-amino-6-deoxy-futalosine, which contains a carboxylic acid group instead of the fluorosulfonyl group on the benzoyl ring. The 6-amino-6-deoxy-futalosine is a native substrate for MTAN, produced in the menaquinone pathway in some bacteria (but not evident in *Borrelia*). Based on its similarity to FSBA, our results suggest that 6-amino-futalosine would also be poorly catalyzed by the *Borrelia* enzymes, although its binding to the enzyme active site would be relatively tight. Interestingly, FSBA is considered an ATP analog, and has been used extensively to study ATP-binding proteins and kinases, where it can act as a suicide substrate and create covalent adducts that inactivate the enzyme active site [56]. However, FSBA activity as a nucleosidase inhibitor appears to be strictly competitive and overcome by excessive amount of substrate. All three of the nucleosides (MSeA, PurTA, and FSBA) demonstrated bacteriostatic effects on *B. burgdorferi* cultures at all tested concentrations, which were readily overcome by subculture in fresh BSKII-RS medium.

The nucleoside IADO contains a 5′ iodine atom that resembles the sulfur atom in the methylthio group, as it also has an unshared outer electron pair that can act as a Lewis base [57]. In spite of this, IADO was found to be a universally poor substrate for *B. burgdorferi* nucleosidases, with 3–6% of the activity found for MTA. The Ki values for IADO are submicromolar to low micromolar and closely resemble those found for PurTA and FSBA. However, IADO was the most effective antimicrobial among the tested inhibitors, and it exhibited bactericidal rather than bacteriostatic activity (Figure 4). Potentially this improved antimicrobial activity relative to other compounds (PurTA and FSBA) may reflect increased transport of IADO into the cell that allows it to inhibit the cytoplasmic Pfs, but there is no concrete evidence for this mechanism at this stage.

The fifth compound examined in this study was the nonhydrolysable transition state nucleoside analog, dEt-ImmA. Our prior studies on this compound showed that it is a very tight binding inhibitor with an affinity for the active site that is approximately 1000-fold better than the other compounds that were examined here. The dEt-ImmA is a tight but slow onset inhibitor that mimics the early and highly dissociative oxocarbenium transition state of the normal substrate as it is hydrolyzed by the enzyme [46]. The catalytic mechanism in a normal enzyme reaction starts with a proton donation to the substrate at the N^7^ atom of the adenine base, enabling the formation of a hydrogen bond between the N^7^ atom and the enzyme. The purine adenine is electron withdrawing, pulling electrons from the O^4^ atom of the ribose sugar, and leading to elongation of the glycosidic bond present between the N^9^ of purine adenine and the C^1^ of ribose sugar and formation of an oxocarbenium ion transition state structure. An S_N_1 attack by an activated nucleophilic water molecule on the C^1^ atom completes the reaction, yielding the ribose sugar and purine adenine [58]. Even though live spirochetes were observed after treatment with 0.4 µM of dEt-ImmA, their functional activity appeared impaired. Possibly this indicates that the native substrates MTA, SAH, and 5′dADO have accumulated; thus, inhibiting *B. burgdorferi* growth. Alternatively, the persistent MTAN inhibition may result in a loss of adenine salvage that leads to purine starvation of the spirochetes over time and eventual cell death. Interestingly, the concentrations of dEt-ImmA required to exert an antimicrobial effect are far larger than the concentrations required for inhibiting MTAN activity, an observation that is consistent with our prior work with similar transition state analogs [18]. This may suggest that the enzyme concentration in the bacteria is sufficiently large that a higher level of analog is necessary to meet the requirement of stoichiometric levels of drug to ensure all active sites can be filled with inhibitor, or that the structure of the inhibitor reduces its transport into viable cells, limiting its impact on cytoplasmic Pfs.

## 5. Conclusions

We demonstrate here that the use of luciferase expressing strains has added value for evaluation of the efficacy of novel antimicrobials against microbes like *B. burgdorferi*, where solid phase plating-based methods on agar used in other bacteria are not effective. From the standpoint of enzyme inhibition, the most effective compound was dEt-ImmA, which showed a far superior affinity for the enzyme active site based on its resemblance to the transition state. However, it was not more active than IADO as a bactericidal agent by *in vitro* culture assays of spirochete viability, suggesting complex mechanisms may ultimately be responsible for causing death of the *B. burgdorferi* cells, and more than one mechanism may be involved. Compounds like IADO are known substrates for human MTA phosphorylase, and the 5-iodo-ribose-1-phosphate product of the reaction has been proposed to be the active metabolite in mammalian cytotoxicity [52]. Our studies did not support IADO as a substrate for the MTANs, so the mechanism of cytotoxicity is not the same as observed in mammalian cells. Rather, the effects of IADO may reflect inhibition of surface/extracellular enzyme (Bgp/MtnN) that may interrupt their function in supplying readily transportable (and metabolically valuable) adenine to the cells. Cellular uptake of IADO and subsequent inhibition of cytoplasmic Pfs may have less impact on purine salvage but impose the intracellular accumulation of growth inhibitory nucleosides that have broad effects in slowing metabolism. While much work remains to be done to more thoroughly understand the mechanism(s) of action of MTAN inhibitors, the studies reported here suggest that MTAN inhibition is a useful target for the development of anti-*B. burgdorferi* antibiotics and additional research in this area is warranted.

## Figures and Tables

**Table 1:** *Borrelia* MTAN specific activities for nucleoside substrates and analogs.

	Specific Activity (U/mg) *			% Max Specific Activity **		
	Enzyme			Enzyme		
Nucleoside	Bgp	MtnN	Pfs	Bgp	MtnN	Pfs
MTA	1.00	1.00	1.70	100	100	100
MSeA	1.79	1.50	0.27	179	150	16
IADO	0.04	0.03	0.11	4	3	6
PurTA	0.14	0.04	0.04	14	4	2
FSBA	0.12	0.02	0.02	12	2	1

* 1 U = 1 µmol MTA hydrolyzed per minute. ** Relative to MTA as a substrate.

**Table 2:** *Borrelia* MTAN kinetic constants for nucleoside substrates and analogs.

	Enzyme		
Nucleoside	Bgp	MtnN	Pfs
MTA *	Km = 0.49 ± 0.10 µM	Km = 9.09 ± 1.62 µM	Km = 0.61 ± 0.16 µM
MSeA	Km = 1.62 ± 0.10 µM	Km = 1.50 ± 0.14 µM	Km = 2.12 ± 0.24 µM
IADO	Ki = 0.63 ± 0.12 µM	Ki = 2.47 ± 0.46 µM	Ki = 0.90 ± 0.07 µM
PurTA	Ki = 1.31 ± 0.18 µM	Ki = 2.05 ± 0.18 µM	Ki = 0.69 ± 0.09 µM
FSBA	Ki = 0.34 ± 0.03 µM	Ki = 2.04 ± 0.13 µM	Ki = 0.39 ± 0.07 µM
dEt-ImmA *	Ki = 2.48 ± 0.38 nM, Ki’ = 0.35 ± 0.02 nM	Ki = 1.48 ± 0.17 nM Ki’ = 0.58 ± 0.05 nM	Ki = 10.49 ± 2.01 nM Ki’ = 0.53 ± 0.15 nM

* Values reported previously in Cornell et al., 2020 [18]. Km = Michaelis constant, Ki = inhibition constant, and Ki’ = delayed onset inhibition constant.

**Table 3:** Measurement of bioluminescence to determine the levels of live *B. burgdorferi.*

*B. burgdorferi*/mL	Equation	R^2^
1.0 × 10^8^	−0.5296x + 39.133	0.9188
1.5 × 10^8^	−0.7453x + 27.088	0.9237
2.0 × 10^8^	−0.7379x + 101.38	0.9834

**Table 4:** Bacteriostatic versus bactericidal activities of MTAN inhibitors.

Nucleoside Inhibitor	Inhibitor Activity	Dose
MSeA	Bacteriostatic	All doses
IADO	Bactericidal	2 µM, 10 µM
PurTA	Bacteriostatic	All doses
FSBA	Bacteriostatic	All doses
dEt-ImmA	Bactericidal	2 µM, 10 µM
